# Overexpression of SKP2 Inhibits the Radiation-Induced Bystander Effects of Esophageal Carcinoma

**DOI:** 10.3390/ijerph14020155

**Published:** 2017-02-06

**Authors:** Xiao-Chun Wang, Tie-Jun Zhang, Zi-Jian Guo, Chang-Yan Xiao, Xiao-Wen Ding, Fang Fang, Wen-Tao Sheng, Xu Shu, Jue Li

**Affiliations:** 1 The Beijing Prevention and Treatment Hospital of Occupational Disease for Chemical Industry (Beijing Institute of Occupational Disease Prevention and Treatment), Beijing 100093, China; wxc3188@126.com (X.-C.W.); xiaochangyan11@163.com (C.-Y.X.); dingxiaowen00@sohu.com (X.-W.D.); fangfang001230@126.com (F.F.); shengwentao112@126.com (W.-T.S.); 2 Department of Nuclear Medicine, The First Affiliated Hospital of Baotou Medical College, Baotou 014010, China; zhangtiejun011@126.com; 3 The First Affiliated Hospital of Guangxi University of Chinese Medicine, Nanning 530023, China; guozijian1101@163.com; 4 National Institute for Radiological Protection, China CDC, Beijing 100088, China

**Keywords:** SKP2, esophageal carcinoma, radiation-induced bystander effect

## Abstract

Background: To investigate the effects of S-phase kinase protein 2 (SKP2) expression on the radiation induced bystander effect (RIBE) in esophageal cancer (EC) cells. Materials and Methods: Western blot was used to detect the levels of SKP2, Rad51, and Ku70 in EC cells. Positive transfection, RNAi, micronucleus (MN), and γ-H2AX focus formation assay were used to investigate the effects of SKP2 on RIBE induced by irradiated cells. Results: We found a significant negative correlation between SKP2 expression and MN frequency (*p* < 0.05) induced by RIBE. The results were further confirmed by positive transfection, RNAi, and rescue experiments.γ-H2AX focus formation assay results indicated that overexpression of SKP2 in the irradiated cells inhibited the DNA damage of RIBE cells. However, when SKP2 expression decreased in irradiated cells, the DNA damage of RIBE cells increased. Increased or decreased expression levels of SKP2 had effects on Rad51 expression under the conditions of RIBE. Conclusions: These results showed, for the first time, that SKP2 expression can inhibit RIBE of EC cells. The mechanism may function, at least partly, through the regulation of Rad51 in the ability to repair DNA damage.

## 1. Introduction

The radiation-induced bystander effect (RIBE) is an interesting biological event in which ionizing radiation can induce cellular damage, both in directly-exposed cells and in their neighboring, non-irradiated cells. Since it was first discovered by Nagasawa [1], RIBE has become the focus of research regarding tumor radiotherapy and radiobiological studies. A series of cellular responses in RIBE have been reported, including micronucleus formation, genomic instability, sister chromatid exchanges, carcinogenesis, and cell killing. Many bystander signal factors, such as NO, TGF-β1, ROS, p53, and IGF, play important roles in RIBE via direct communication or culture medium transmission [2,3].

RIBE has important implications in tumor radiotherapy. A variety of bystander effects have been found in bystander cells, co-cultured with irradiated tumor cells. The study of hepatoma cells showed that irradiated cells could induce bystander effects in co-cultured, non-irradiated cells via the p53 pathway [4,5]. On the other hand, evidence has shown that bystander normal human fibroblast cells reduced damage response in irradiated human melanoma cells through intercellular ROS level modulation [6]. In contrast, the radio-sensitivity of breast cancer cells could be enhanced by bystander fibroblast cells via the Akt pathway [7]. Hence, RIBE is significant to clinical tumor radiotherapy.

S-phase kinase protein 2 (SKP2) is the substrate recognition subunit of the SCF^SKP2^ ubiquitin ligase complex, and was originally identified as a protein that interacts with cyclin A [8,9]. This protein is implicated in ubiquitin-mediated degradation of the cyclin dependent kinase (CDK) inhibitor p27^KIP1^, and positively regulates G1/S transition. Targeted inactivation of the SKP2 gene results in the accumulation of p27 and cyclin E, and causes the cell cycle to be blocked in the G1 phase. SKP2 also plays an important role in regulating the cell cycle by controlling the expression of other target proteins, including p21, p57, p130, c-Myc, and E2F1, which are associated with the initiation, development, treatment, and prognosis of cancer [10]. SKP2 is an oncogene and is overexpressed in many human tumors in different organs; examples of such cancers are esophageal, prostate, breast, stomach, and colon [11,12,13,14]. However, until now, no reports regarding the effects of SKP2 on the bystander response of esophageal cancer (EC) have been found. In the present study, we found, for the first time, that overexpression of SKP2 inhibited the bystander effects of EC.

## 2. Materials and Methods

### 2.1. Plasmid Construction and Small Interfering RNA Synthesis

The SKP2 siRNA, and the control siRNA (a scrambled sequence without significant homology to human gene sequences), were both purchased from Santa Cruz Biotechnology (Shanghai, China). The pcDNA3. 1-SKP2 overexpression vector and mutant pcDNA3.1-SKP2 vector (preserving the same amino acid sequence, but containing three-point mutations in the target nucleotide sequence, and, therefore, resistant to SKP2-RNAi) were produced by GeneChem (Shanghai, China).

### 2.2. Cell Culture and Transfection

Four esophageal carcinoma cell lines (EC9706, KYSE150, KYSE510, and KYSE450) were obtained from Professor Wang (the Cancer Institute (Hospital), Peking Union Medical College and Chinese Academy of Medical Sciences). Cells were cultured in RPMI 1640 medium (Invitrogen, Carlsbad, CA, USA) supplemented with 10% FBS (Gibco, Carlsbad, CA, USA) at 37 °C under 5% CO_2_ in a humidified incubator. Cell transfections were performed using a Lipofectamine 2000 (Invitrogen, Carlsbad, CA, USA), according to manufacturer’s instructions. In transient transfections, 100 nM siRNA (SKP2 siRNA or scrambled siRNA) was used, and cells were harvested for subsequent experiments 48 h after transfection. In the stable transfection, cells were selected with 200 μg/mL G418 and the pool of SKP2 overexpression was chosen for subsequent experiments. Western blot was used to confirm transfections results.

### 2.3. Western Blot Analyses

Cultured cells were harvested and treated with a lysis buffer (10 mM Tris-HCl (pH 7.5), 150 mM NaCl, 1% NP40) containing a cocktail of protease inhibitors (Invitrogen, Carlsbad, CA, USA). After centrifugation at 12,000 rcf for 10 min at 4 °C, supernatants were collected. The cell lysate was kept at 95 °C for 10 min. The protein was separated using SDS-PAGE, and was transferred to a polyvinylidenedifluoride (PVDF) membrane (Millipore, Shanghai, China). The membrane was blocked with 5% fat-free dry milk in TBST (containing 0.1% Tween-20) for 1 h at room temperature. Then, the membrane was incubated for 18 h at 4 °C with antibodies against SKP2, Rad51, and Ku70 (1:500 dilution, Santa Cruz Biotechnology, Shanghai, China). After being washed three times with TBST, the membrane was incubated with horseradish peroxidase-conjugated secondary antibody with a 1:5000 dilution (Cell Signaling Technology, Shanghai, China) and visualized with the ChemiDoc XRS system (Bio-Rad Laboratories, Hercules, CA, USA) using super ECL detection reagent (Applygen, Beijing, China).

### 2.4. Irradiation and Co-Culture

EC cells were exposed to different doses of γ-ray radiation in a J. L. Shepherd Model 143 ^137^Cs irradiator (J. L. Shepherd & Associates, San Fernando, CA, USA) at a dose rate of 2.4 Gy/min. After radiation, the irradiated cells and the non-irradiated cells were plated in different coverslips and were placed, face-to-face, with a 3-mm gap in a 35 mm dish. After 24 h of co-culture, the cells were harvested for the following experiments.

### 2.5. MN Assay

The cells were treated with 1 μg/mL cytochalasin B (Sigma, St. Louis, MO, USA) for 28 h. Then, the cells were harvested and treated with a hypotonic solution (0.075 M KCl). After immediate centrifugation at 1000 rcf for 8 min at 4 °C, the cells were fixed, in situ, with methanol/acetic acid (3:1 v/v) for 30 min. Air-dried cells were stained with 0.01% Giemsa stain (Sigma, St. Louis, MO, USA) for 5 min and then observed under a microscope. MN was observed in at least 500 binucleated cells, and the MN yield was calculated as the ratio of the number of MN to the counted number of binucleated cells.

### 2.6. γ-H2AX Focus Formation Assay

The cells were grown on coverslips that were kept in 35-mm dishes overnight, and irradiated with 0 and 8 Gy γ-ray, respectively. Cells were harvested and washed with PBS. Then, cells were fixed in 4% paraformaldehyde for 20 min at room temperature. Next, the cells were washed twice with PBS. For immunofluorescence staining, cells were permeabilized for 3 min in 0.25% Triton X-100 in PBS, washed twice with PBS, and blocked for 1 h with 5% BSA in PBS at room temperature. Antibodies were diluted (1:200) in 1% BSA in PBS. Cells were incubated with primary antibodies for 1.5 h at room temperature, washed three times in PBS, and then incubated with secondary antibodies for 1 h at room temperature. Finally, cells were rinsed and mounted with ProLong Gold antifade with a DAPI mounting medium (Molecular Probes, Eugene, OR, USA). Images were captured using a Carl Zeiss confocal microscope (Carl Zeiss AG, Jena, Germany). Acquisition settings were optimized to obtain the maximal signal in immunostained cells with minimal background. Two hundred nuclei were counted. All experiments were carried out in triplicate, independent of each other. The primary antibody used was rabbit anti-γ-H2AX (Cell Signaling, Danvers, MA, USA); the secondary antibody used was Alexa Fluor 488 goat anti-rabbit IgG (Invitrogen, Carlsbad, CA, USA).

### 2.7. Flow Cytometry

Cells were harvested for 8 h, post irradiation, with doses of 0 and 8 Gy for apoptosis detection using the annexin V-FITC apoptosis detection kit (Sigma, St. Louis, MO, USA), and were subsequently analyzed by flow cytometry. 

### 2.8. Statistical Analyses

All statistical analyses were performed using SPSS 16.0 software (SPSS Inc., Armonk, NY, USA). Experimental results were obtained from three independent experiments and evaluated using a χ^2^ test and ANOVA. *p* < 0.05 was determined being statistically significant.

## 3. Results

### 3.1. Endogenous SKP2 Protein Levels in Four EC Cell Lines

**Figure 1:**
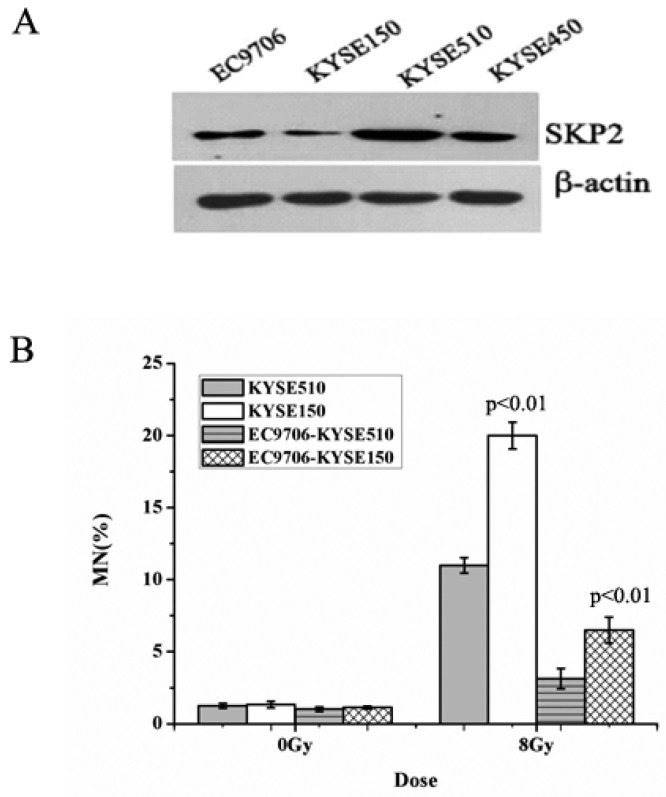
Effects of endogenous SKP2 expression on the RIBE of esophageal carcinoma (EC) cells. (**A**) SKP2 expression in four EC cells. The sequence of SKP2 levels was KYSE510 > KYSE450 > EC9706 > KYSE150. β-actin was used as an internal control; (**B**) MN formation in γ-ray irradiated EC cells (KYSE510, KYSE150) and bystander EC cells (EC9706). MN yields of 8-Gy-irradiated KYSE150 were significantly higher than those of irradiated KYSE510 (*p* < 0.01). As the bystander cell, EC9706 co-cultured with irradiated KYSE150 had higher MN yields than EC9706 co-cultured with irradiated KYSE510 (*p* < 0.01). Results correspond to the mean ± SD of experiments, performed in triplicate, in each case.

The expression of SKP2, in four EC cells lines, was measured using Western blot. As shown in Figure 1A, the sequence of SKP2 levels was KYSE510 > KYSE450 > EC9706 > KYSE150. Then, the MN assay was performed in order to determine the relationship between RIBE and SKP2. The MN yields of irradiated KYSE510 with a high level of SKP2 were lower than those of irradiated KYSE150 with a low level of SKP2 (*p* < 0.01) (Figure 1B). Meanwhile, the bystander non-irradiated EC9706, co-cultured with irradiated KYSE510, showed a lower MN frequency than bystander cells co-cultured with irradiated KYSE150 (*p* < 0.01) (Figure 1B). These results indicated that SKP2 expression could inhibit RIBE induced by irradiated EC cells.

### 3.2. Relationship between RIBE and Overexpression of SKP2

**Figure 2:**
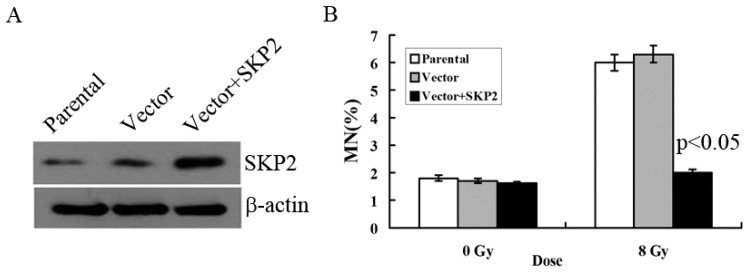
Effects of SKP2 overexpression on RIBE of EC cells. (**A**) Compared with the control groups, SKP2 expression in KYSE150 cells increased after positive transfection; (**B**) The MN assays results showed that the EC9706 cells co-cultured with positive SKP2 transfection cells had lower MN yields than the parental and control cells (*p* < 0.05). Results correspond to the mean ± SD of experiments, performed in triplicate, in each case.

We constructed the overexpression vector of SKP2 and positively transfected it to EC9706 cells in order to detect the effects of SKP2 overexpression on RIBE. The parental cell line and empty vector were used as control. Compared with the control groups, Western blot results showed that the expression level of SKP2 increased after positive transfection, showing that the overexpression vector of SKP2 was function (Figure 2A). Then, MN assay results showed that the MN frequency in bystander EC9706 cells, co-cultured with transfected KYSE150 cells (with increased SKP2 expression), was lower than that of the control groups (*p* < 0.05) (Figure 2B).

### 3.3. Relationship between RIBE and Decreased SKP2 Expression

**Figure 3:**
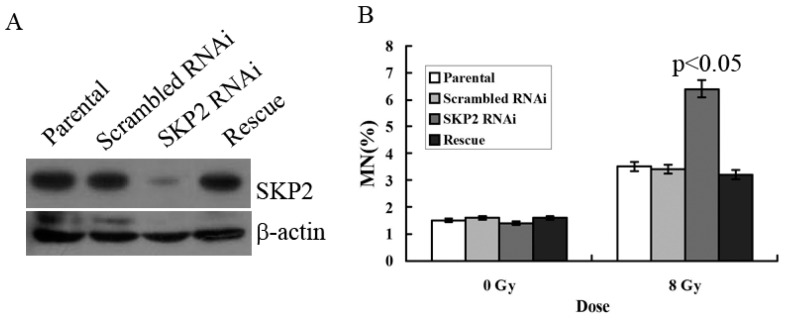
(**A**) Expression of SKP2 in KYSE510 cells decreased after SKP2 knockdown, yet increased in rescue groups; (**B**) MN assay showed that EC9706, co-cultured with SKP2 RNAi cells, had higher MN yields than the parental and scrambled RNAi cells (*p* < 0.05). However, in the rescue group, the MN frequency was also significantly lower than that of RNAi cells. Results correspond to the mean ± SD of experiments, performed in triplicate, in each case.

To verify the effects of SKP2 on RIBE, we constructed the RNAi and rescue vector of SKP2 in order to examine the effects of SKP2 expression on the MN assay. The parental and scrambled RNAi were used as control. Compared with the control groups, Western blot results showed that the expression level of SKP2 decreased after knockdown, but the SKP2 level was rescued after transfection rescue vector, showing that these two vectors (RNAi and rescue) were all functional (Figure 3A). MN assay results showed that the MN frequency in bystander EC9706 cells co-cultured with SKP2 knockdown KYSE510 cells was higher than those of the control groups *(p <* 0.05). However, in the rescue group cells, the MN frequency was also significantly lower than that of RNAi cells (*p <* 0.05) (Figure 3B). Thus, the results proved that changes of SKP2 level have effects on the results of RIBE.

### 3.4. Effects of SKP2 Expression on the Repair of DSBs Induced by RIBE

**Figure 4:**
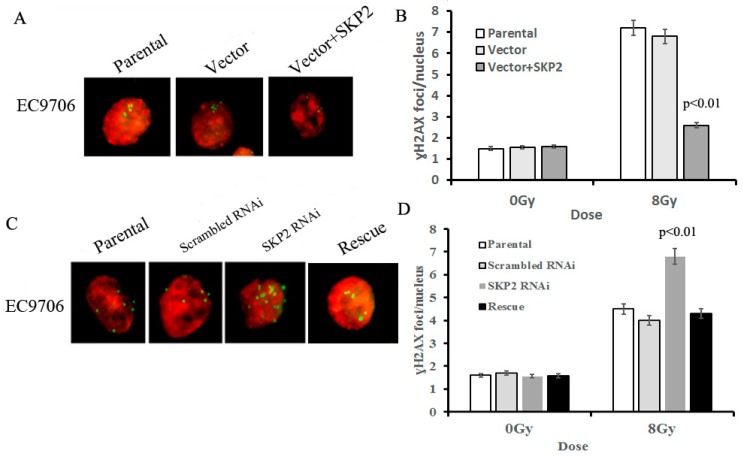
Effects of SKP2 expression on the repair of DSBs induced by RIBE. (**A**) γ-H2AX foci in nucleus of EC9706 cells co-cultured with parental, vector, and vector + SKP2 irradiated KYSE150 cells; (**B**) The number of γ-H2AX foci per nucleus in EC9706 co-cultured with induced SKP2 cells was significantly lower than that in EC9706 co-cultured with control cells (*p* < 0.01); (**C**) γ-H2AX foci in nucleus of EC9706 cells co-cultured with parental, scrambled RNAi, SKP2 RNAi, and SKP2 rescue irradiated KYSE510 cells; (**D**) The number of γ-H2AX foci per nucleus in EC9706 co-cultured with SKP2 knockdown cells was significantly higher than that in EC9706 co-cultured with control cells (*p* < 0.01). Conversely, the number of γ-H2AX foci per nucleus in EC9706 co-cultured with SKP2 rescued cells was decreased when compared with the SKP2 RNAi group. In the γ-H2AX focus formation assay, two hundred nuclei were analyzed per experiment. The γ-H2AX focus and nucleus was stained with Cy3 and DAPI, respectively. Results correspond to the mean ± SD of experiments, performed in triplicate, in each case.

The γ-H2AX focus formation assay was used to determine the effects of different SKP2 levels on the damage to DNA, induced by RIBE. The amount of γ-H2AX foci in bystander cells co-cultured with SKP2 positive transfected cells was significantly lower than those of the control groups (*p* < 0.01) (Figure 4A,B). Conversely, the number of γ-H2AX foci in bystander cells co-cultured with SKP2 knockdown cells was significantly higher than those of the control groups (*p* < 0.01). However, the number of γ-H2AX foci in the rescue group was significantly lower than that of the RNAi group (*p* < 0.01) (Figure 4C,D). These data showed that SKP2 expression could affect the repair ability of DSBs induced by RIBE.

### 3.5. Effects of SKP2 Expression on DSBs Repair Relative Proteins

**Figure 5:**
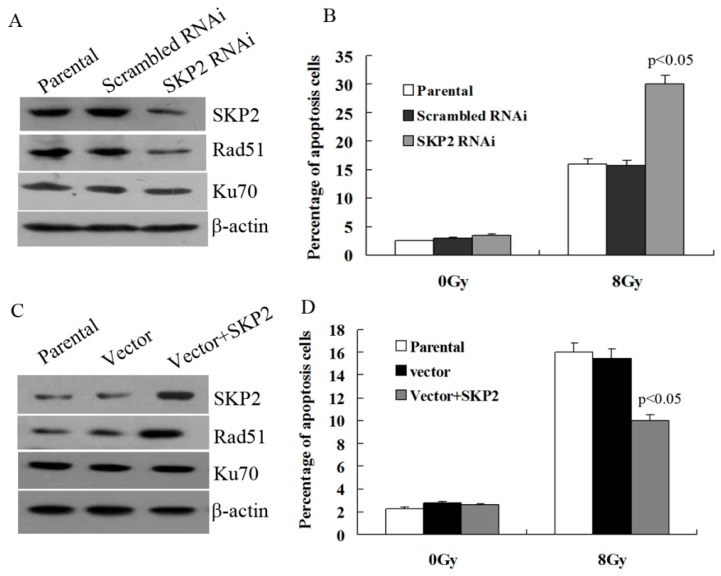
Effects of SKP2 on Rad51, Ku70 expression, and apoptosis of RIBE cells. (**A**) Down-regulation of SKP2 in irradiated KYSE510 cells led to a decreased expression of Rad51 in bystander EC9706 cells. No changes in Ku70 expression were observed; (**B**) The percentage of apoptosis EC9706 cells co-cultured with KYSE510 cells with a low SKP2 level was significantly higher than those of the control cells (*p* < 0.05); (**C**) Up-regulation of SKP2 in irradiated KYSE150 cells led to increased expression of Rad51 in bystander EC9706 cells. No changes in Ku70 expression were observed; (**D**) The percentage of apoptosis EC9706 cells co-cultured with KYSE150 cells with a high SKP2 level was significantly lower than those of the control cells (*p* < 0.05). Results correspond to the mean ± SD of experiments, performed in triplicate, in each case.

Next, we explored whether SKP2 has an effect on the expression of key DSBs repair proteins, Rad51 and Ku70. As shown in Figure 5A, the expression of SKP2 in KYSE 510 cells decreased after RNAi experiments, and Rad51 expression decreased after SKP2 knockdown in RIBE EC9706 cells. However, the Ku70 level did not change. According to this result, the percentage of apoptosis EC9706 cells co-cultured with irradiated KYSE510 cells, with a low SKP2 level, was significantly higher than those of the control cells (*p* < 0.05) (Figure 5B). Forced expression of SKP2in KYSE150 cells contributed to the expression of Rad51 in co-cultured EC9706 cells, and Ku70 expression was not affected (Figure 5C). The percentage of apoptosis EC9706 cells co-cultured with irradiated KYSE 150 cells with a high SKP2 level was significantly lower than those of the control cells (*p* < 0.05) (Figure 5D).

## 4. Discussion

Previous studies have found some signal factors that could regulate RIBE in various types of human tumors. Shao et al. demonstrated that RIBE could be modulated by the p53 status of irradiated hepatoma cells, and a p53-dependent release of cytochrome-c was involved in RIBE [4]. Further research proved that cytochrome-c regulated the bystander response through an iNOS-triggered NO signal. Additionally, another group found that ROS played an important role in mediating RIBE via a p53-dependent SCO2 pathway in hepatoma cells [2]. Temme et al. found that enhanced superoxide anion production was substantially reduced prior to the release of the bystander signal, activated by TGF-β in human gastric cancer cells [3]. However, up to now, there is almost no research regarding the relationship between SKP2 and RIBE in human cancers. Evidence shows that SKP2 had a high expression level in several human tumors and could promote the proliferation and metastasis of cancer cells. Our previous study showed that overexpression of SKP2 suppressed anoikis and promoted metastasis of EC. Moreover, we have demonstrated that increased SKP2 expression improved the radio-resistance of EC cells [15]. Based on our previous studies, we assumed SKP2 to have effects on the RIBE of EC cells. Thus, we first examined the expression of SKP2 in four EC cells lines, and found that the SKP2 level was highest in KYSE 510 and lowest in KYSE150 cells. Given that the formation of micronuclei is a hallmark of genotoxicity, the micronucleus (MN) assay was performed in order to determine the relationship between RIBE and SKP2 expression. The MN yields of irradiated KYSE510 with a high level of SKP2 were lower than those of irradiated KYSE150 with a low level of SKP2. Meanwhile, bystander EC9706 co-cultured with irradiated KYSE510 showed a lower MN frequency than that of bystander cells co-cultured with irradiated KYSE150. These results indicated that SKP2 expression could inhibit RIBE induced by irradiated EC cells. To further confirm these results, we constructed the overexpression, RNAi, and rescue vector of SKP2, respectively, and performed a series of experiments. MN assay showed that the bystander MN frequency was reduced in non-irradiated EC cells after being co-cultured with irradiated EC cells with a high SKP2 level. Conversely, low expression of SKP2 after knockdown promoted RIBE, and this effect was rescued by the SKP2 rescue vector. All these results proved that SKP2 expression has effects on the RIBE of EC cells. Given that RIBE has important implications for secondary cancer risk assessment during cancer radiotherapy, SKP2 may be a potential target for EC radiotherapy.

Ionizing radiation always induces DNA damage and the ability to repair DNA DSBs is one of the most important factors influencing the survival of cells that are directly or indirectly affected by radiation. If the damage induced by radiation can be efficiently repaired, the cell will survive, otherwise, it will undergo apoptosis. Our previous study regarding radio-sensitivity showed that overexpression of SKP2 could promote the ability to repair DSB induced by radiation in EC9706 cells. Thus, we hypothesized that SKP2 affects the ability to repair DSBs induced by RIBE. In the present study, the γ-H2AX focus formation assay supported the observation of the effects of SKP2 on the damage to DSBs in RIBE. There are several key factors in the pathways of repairing DSBs, including Rad51, p27, Ku70, and Akt protein [16,17]. Galanos et al. showed that, under persistent DNA damage, Rad51 can be exhausted, allowing Rad52 to guide DNA repair in an error-prone manner, which eventually leads to genomic instability [18]. We examined the effects of SKP2 expression on Rad51, Ku70 expression, and apoptosis, respectively. Results showed that, with the changes of SKP2 expression in irradiated cells, the Rad51 level also changed in bystander EC cells. No changes in Ku70 expression were observed. The expression of SKP2 in irradiated cells also had effects on the percentage of apoptosis in bystander EC cells. These results showed that the expression of SKP2 has effects on the damage and repair of RIBE cells *via* the Rad51 pathway. However, the manner in which SKP2 affects the repair of DNA damage in bystander cells is still unknown. We will explore this in future work.

## 5. Conclusions

Our results revealed, for the first time, that overexpression of SKP2 decreased micronucleus frequency and promoted the ability to repair DSBs of bystander non-irradiated EC cells. Therefore, overexpression of SKP2 could inhibit RIBE induced by irradiated EC cells, and SKP2 may be a potential target for EC radiotherapy.
